# Preoperative intra-aortic balloon pump improves the clinical outcomes of off-pump coronary artery bypass grafting in left ventricular dysfunction patients

**DOI:** 10.1038/srep27645

**Published:** 2016-06-09

**Authors:** Feng Yang, Jinhong Wang, Dengbang Hou, Jialin Xing, Feng Liu, Zhi chen Xing, Chunjing Jiang, Xing Hao, Zhongtao Du, Xiaofang Yang, Yanyan Zhao, Na Miao, Yu Jiang, Ran Dong, Chengxiong Gu, Lizhong Sun, Hong Wang, Xiaotong Hou

**Affiliations:** 1 Center for Cardiac Intensive Care, Beijing Institute of Heart, Lung and Blood Vessel Diseases, Beijing Anzhen Hospital, Capital Medical University, Beijing, China; 2 Department of Cardiac Surgery, Beijing Institute of Heart, Lung and Blood Vessel Diseases, Beijing Anzhen Hospital, Capital Medical University, Beijing, China

## Abstract

Severe left ventricular (LV) dysfunction patients undergoing off-pump coronary artery bypass grafting (OPCAB) are often associated with a higher mortality. The efficacy and safety of the preoperative prophylactic intra-aortic balloon pump (IABP) insertion is not well established. 416 consecutive patients with severe LV dysfunction (ejection fraction ≤35%) undergoing isolated OPCAB were enrolled in a retrospective observational study. 191 patients was enrolled in the IABP group; the remaining 225 patients was in control group. A total of 129 pairs of patients were propensity-score matched. No significant differences in demographic and preoperative risk factors were found between the two groups. The postoperative 30-day mortality occurred more frequently in the control group compared with the IABP group (8.5% vs. 1.6%, *p* = 0.02). There was a significant reduction of low cardiac output syndrome in the IABP group compared with the control group (14% vs. 6.2%, *p* = 0.04). Prolonged mechanical ventilation (≥48 h) occurred more frequently in the control group (34.9% vs. 20.9%, *p* = 0.02). IABP also decreased the postoperative length of stay. Preoperative IABP was associated with a lower 30-day mortality, suggesting that it is effective in patients with severe LV dysfunction undergoing OPCAB.

Coronary artery disease (CAD) is the most common underlying disease for left ventricular (LV) dysfunction among adults in China and many other countries^1^,^2^. The number of CAD patients with LV dysfunction continues to increase^3^. Many studies have shown a favorable survival benefit in patients undergoing surgical revascularization^4^,^5^. Off-pump coronary artery bypass graft (OPCAB) surgery may achieve comparable in-hospital outcomes and is now generally performed in some developing countries, such as China and India^6^,^7^,^8^. However, severe LV dysfunction (ejection fraction ≤35%) is reported to be the predictor of mortality in OPCAB^5^. In clinical practice, patients with severe LV dysfunction have generally been excluded from OPCAB procedures by some cardiac surgeons because of the potential risk for hemodynamic deterioration and conversion to on-pump coronary artery bypass graft (CABG)^9^.

Intra-aortic balloon pump (IABP) can not only maintain blood hemodynamic stability during OPCAB procedures^10^, but also can increase coronary artery blood infusion in percutaneous cornary interventions (PCI)^11^. Although previous reports have shown conflicting results for preoperative IABP insertion in high-risk patients undergoing surgical revascularization^12^,^13^,^14^,^15^,^16^,^17^,^18^,^19^,^20^, the evidence from several meta-analyses support preoperative IABP insertion for high-risk patients^12^,^14^,^17^. Unfortunately, these meta-analysis studies included few OPCAB patients. Therefore, we retrospectively investigated the data of preoperative IABP insertion in patients with severe LV dysfunction that underwent OPCAB and performed a propensity-matched analysis to determine whether these patients benefited from preoperative prophylactic IABP insertion.

## Material and Methods

### Patient population

**Figure 1:**
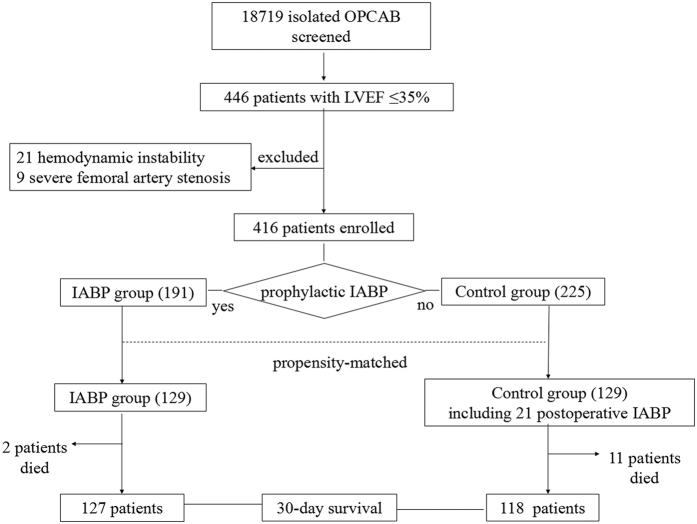
Study flow. A total of 18,719 patients undergoing isolated OPCAB were screened. The LVEF of 446 patients was less than 35%, and 416 patients were enrolled. Prophylactic IABP was inserted in 191 patients (IABP group); 225 patients did not receive prophylactic IABP (control group); 258 patients (129 patients in each group) were propensity matched. The postoperative 30-day mortality and morbidity were compared. IABP denotes intra-aortic balloon pump, LVEF denotes left ventricular ejection fraction, and OPCAB denotes off-pump coronary artery bypass graft.

The present study was a historical, single-center, observational cohort study conducted at Beijing Anzhen Hospital, Capital Medical University. A total of 18,719 consecutive adult patients underwent isolated OPCAB in this hospital from January 2009 to December 2014. The left ventricular ejection fraction (LVEF) of all patients was calculated from an echocardiography assessment performed before the OPCAB operation. A total of 446 (2.4%) patients were identified as having severe LV dysfunction (LVEF ≤ 35%). Among those patients, 30 cases that presented with hemodynamic instability or severe femoral artery stenosis were excluded (Fig. 1). Finally, 416 patients were categorized according to preoperative prophylactic IABP insertion into either the IABP group or the control group. Data were retrospectively extracted from an institutional registry of OPCAB patients and ICU clinical database. This study received approval from the Beijing Anzhen Hospital Research Ethics Committee and all operations were performed in accordance with relevant guidelines and regulations. Because this was a retrospective observational study, the institutional review board of the Beijing Anzhen Hospital Ethics Committee waived informed consent. Data collection was subjected to the supervision of the Beijing Municipal Administration of Hospitals.

### Surgical technique

The detailed information of the OPCAB procedure has been previously described^7^. Six experienced cardiac surgeons (≥200 cases/year) performed all of the procedures. A cardiopulmonary bypass (CPB) circuit was placed on stand-by during the procedures. Conversion to CPB was considered if there was any evidence of hemodynamic instability concerns, such as ventricular arrhythmia, hypotension (systolic pressure ≤80 mmHg), and cardiac arrest during OPCAB procedures.

### IABP support

Patients in the IABP group received IABP support prior to the induction of anesthesia, followed by continuous IABP during the entire procedure, and postoperatively if needed. Each patient provided written consent for the IABP insertion. The control patients did not receive IABP support preoperatively. IABP treatment was initiated in the control group when cardiac index (CI) could not be maintained at a level ≥2.0 L/min/m^2^, regardless of a high inotrope status.

Two types of IABP system were used at our center, namely, the Arrow System and the Datascope CS300 System. IABP balloon was selected according to the height of the patients. The IABP balloon was connected to a Datascope or Arrow pump. IABP insertion through the best femoral artery was possible in all cases according to the results of an ultrasound examination of bilateral lower extremities, and the corrected placement was assessed by a chest X-ray.

The IABP variables were settled at a 1:1 balloon inflation synchronized with the electrocardiogram or aortic blood flow. Heparin was systematically used for anticoagulation. IABP support was terminated with hemodynamic stability after the operation.

### Primary and secondary outcomes and definitions

The primary endpoint was postoperative 30-day mortality (death occurring within 30 days after surgery). The secondary endpoints were major postoperative complications, such as low cardiac output syndrome (LCOS), myocardial infarction, bleeding requiring reoperation, tracheotomy, renal failure requiring dialysis, stroke, and ICU stay, and postoperative length of stay (LOS)^21^. The IABP-related complications were also recorded and analyzed for safety purposes.

### Statistical analysis

Continuous variables are shown as the mean and standard deviations or the median and interquartile ranges, and categorical variables are shown as frequencies and percentages. Continuous variables were compared using Student’s t-test or Mann-Whitney U-test. Categorical variables were compared using the Chi-square test or a Fisher’s exact test.

Propensity score matching was undertaken by estimating the probability of receiving preoperative IABP support (that is, the propensity score) using a logistic regression model including the potentially confounding covariates shown in Table 1. Each IABP group patient was matched with a non-IABP-supported patient based on the propensity score by the method of nearest neighbor matching within a caliper (caliper = 0.25 × SD[logitP]^22^) using the psmatch2 command in Stata^23^. After matching, continuous variables that followed a normal distribution were compared using the paired sample *t*-test; otherwise, the Wilcoxon’s matched-pairs signed-rank test was used. The McNemar’s test was used to evaluate the comparability of categorical factors after matching. The in-hospital survival rates after surgery of matched samples were shown as Kaplan-Meier survival cures and the effect of IABP vs. non-IABP was presented as a hazard ratio (HR) with associated 95% confidence intervals (CIs) from the Cox regression model.

Statistical analyses were conducted using Stata software version 11 (Stata Corp, College Station, Texas, USA). Two-sided testing was used with a *p*-value significance level of less than 0.05.

## Results

### Patient baseline characteristics

**Figure 2:**
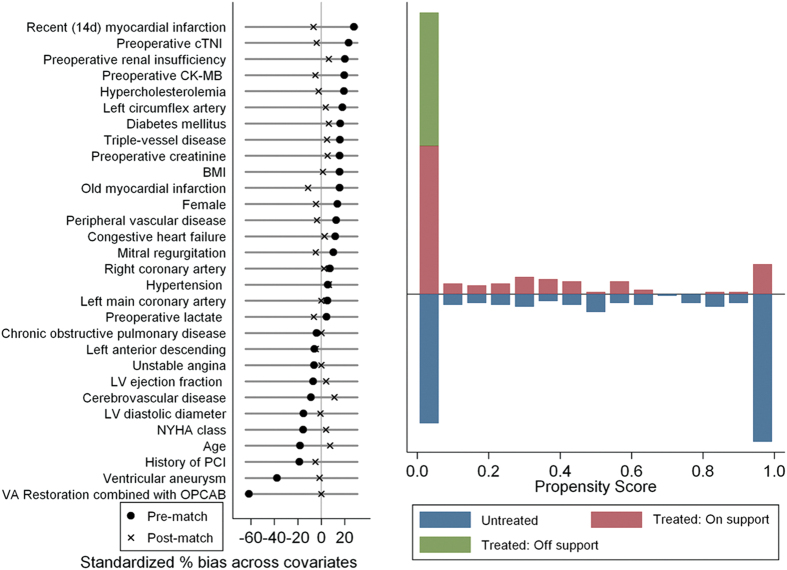
Love (left panel) and Jitter plot (right panel) in propensity-matched analysis.

A total of 416 patients were included in this study (Fig. 1), with 191 patients in the IABP group and 225 patients in the control group. There were significant differences documented in the demographics and comorbidities of the patients (Table 1). After the propensity-score matching, 129 matched pairs were obtained (Fig. 1). No differences in demographics or preoperative risk factors were found between the two groups (Table 1). Figure 2 shows a Love plot for the absolute differences in the baseline covariates before and after matching; a jitter plot for propensity-score distribution is also presented.

OPCAB procedural characteristics are detailed in Table 2. Importantly, the mean number of anastomoses were comparable between the two groups (*p* = 0.22). However, there were few patients converted to on-pump CABG in the IABP group (0 vs. 6 [4.6%], *p* = 0.04).

### Postoperative 30-day mortality

**Figure 3:**
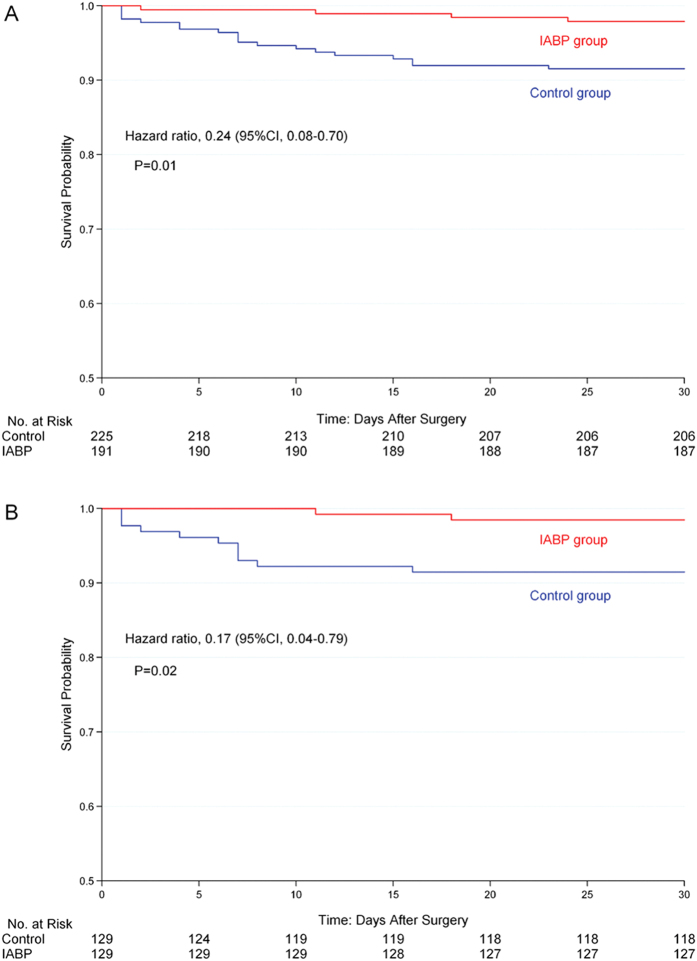
Kaplan-Meier cumulative 30-day mortality after surgery. Kaplan-Meier survival curves shows 30-day mortality in surgical patients with preoperative IABP (red line) and without preoperative IABP (control, blue line) before (**A**) and after (**B**) propensity-score matching. IABP denotes intra-aortic balloon pump.

Clinical outcomes are presented in Table 2. The 30-day mortality was 1.6% in IABP group compared to 8.5% in the control group (*p* = 0.02). The Kaplan-Meier survival curves of the two groups before and after matching are shown in Fig. 3. The preoperative prophylactic IABP insertion was an independent predictor of survival after adjusting for the propensity score using the Cox regression model (HR 0.17, CI 0.04–0.79, *p* = 0.02).

### Postoperative complications

The postoperative complications are summarized in Table 2. There was a significant reduction in postoperative LCOS in the IABP group (14 vs. 6.2%, *p* = 0.039). There were no significant differences in the required transfusion of red blood cells (2 vs. 2 units, *p* = 0.07) and fresh frozen plasma (600 vs. 600 ml, *p* = 0.37). The postoperative myocardial infarction, reoperation for bleeding, tracheotomy, hemodialysis, and neurologic events were comparable between the two groups (Table 2).

There were no statistically significant differences in the duration of mechanical ventilation (47.1 ± 49.1 vs. 37.9  ±  40.9 h, *p* = 0.16). However, the required prolonged mechanical ventilation occurred more frequently in the control group (34.9 vs. 20.9%, *p* = 0.02). ICU stay was comparable between the two groups. The mean postoperative LOS was shorter in the IABP group (interquartile range) of 9 (7–12) vs. 10 (7–16) days (*p* = 0.03) (Table 2).

**Figure 4:**
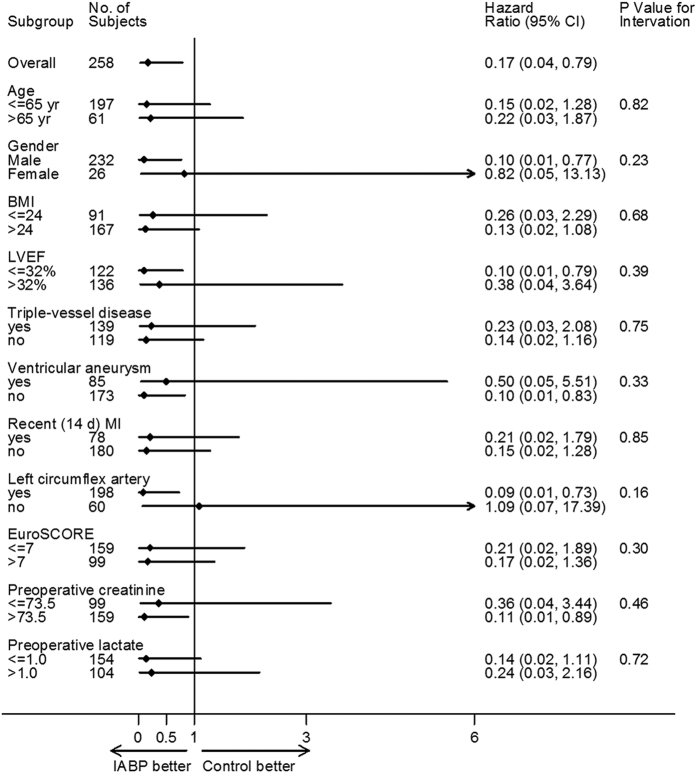
Subgroup analysis for 30-day mortality after surgery. Subgroup analyses are shown for 30-day mortality between patients with preoperative IABP vs. controls. The *p*-value for interaction represents the likelihood of interaction between the variable and the relative treatment effect. BMI denotes body mass index, LV denotes left ventricular, NYHA denotes New York Heart Association, and EuroSCORE denotes European System for Cardiac Operative Risk Evaluation.

No significant interactions were observed between preoperative IABP insertion and any of the 12 sub-groups with respect to the in-hospital mortality, as shown in the hazard-ratio plots in Fig. 4.

### IABP-related complications

Post-cardiotomy IABP was used in 21 patients in the control group, including 3 who required weaning from CPB, and 18 who occurred LCOS. The mean duration of IABP support was shorter in the IABP group (135.9  ±  80.9 vs. 76.4  ±  33.4 h, *p* < 0.001). There were no cases of IABP-related mortality. No severe bleeding at the IABP insertion site, or balloon failure in any of the patients. Lower limb ischemia requiring surgical intervention was observed in 1 patient (0.8%) in the IABP group (Table 2).

## Discussion

This large regional study of propensity-matched patients indicated that preoperative prophylactic IABP insertion was associated with reduced postoperative 30-day mortality in severe LV dysfunction patients who underwent OPCAB. Furthermore, prophylactic IABP insertion resulted in significant reduction of postoperative LCOS incidence and shorter postoperative hospital stay.

From the perspective of pathophysiology, the positive effect of IABP insertion is believed to increase coronary blood flow while simultaneously decreasing myocardial oxygen demand. Consequently, preoperative prophylactic IABP assistance provides better hemodynamic stability in crucial times of higher oxygen demand when the heart is displaced in OPCAB procedures^24^,^25^.

Although there are certain advantages in theory, the results have been controversially debated in clinical practice. Some studies have shown a positive effect of preoperative prophylactic IABP insertion in improving the outcomes of high-risk patients^13^,^14^. The strongest evidence supporting preoperative IABP insertion for high-risk patients undergoing CABG comes from published meta-analysis studies^12^,^14^,^17^. However, many contemporary studies have challenged the effectiveness of preoperative IABP in high-risk patients undergoing CABG^15^,^16^. Worse outcomes were shown in a recent propensity-score matching study^16^, in which the preoperative IABP insertion in patients undergoing CABG after acute myocardial infarction was associated with increased in-hospital morbidity, greater transfusion requirements, and longer postoperative ICU stay.

The results of previous studies have been controversial for several possible reasons. First, there is no standard definition of a high-risk patient. Various conditions, including severe LV dysfunction, left main disease, diffuse coronary disease, and reoperation, have been suggested for preoperative prophylactic IABP insertion^14^. Second, the criteria for prophylactic IABP insertion have not been well defined^26^. A distinction is lacking between therapeutic use for patients with preoperative cardiogenic shock or hemodynamic instability and prophylactic use for patients with preoperative hemodynamic stability. In many previous studies including patients with hemodynamic instability, the indication has been more likely for therapeutic, rather than prophylactic insertion. Third, the results were also possibly affected by the severity of the patients who were selected to receive preoperative IABP support. Finally, most of the procedures were conducted in CABG patients. IABP insertion before surgery was suspended during CPB. The benefit from IABP support was relatively low.

Our study investigated for the clinical effects of preoperative prophylactic IABP insertion in patients with severe LV dysfunction that underwent selective OPCAB. The IABP group of patients received preoperative prophylactic IABP support to increase the safety of OPCAB procedures. The patients who received preoperative IABP support for hemodynamic instability, cardiogenic shock, and emergency operations were excluded. Comparatively, IABP was still working during OPCAB procedures in our study. These patients were more likely to benefit from preoperative IABP insertion. Therefore, preoperative prophylactic IABP insertion was associated with a lower rate of conversion to on-pump CABG, which has been associated with increased in-hospital mortality^9^.

The preoperative prophylactic IABP insertion in our study was not associated with an increased rate of IABP-relationship complications (that is, limb ischemia requiring surgical intervention, severe bleeding at the IABP insertion site, and embolism). The incidence rate of IABP-related complications was low, similar with a previous study^27^. Therefore, IABP insertion is safe in those high-risk patients.

### Limitations

Our study had several limitations. First, this study was subject to the limitations inherent in any retrospective, observational study from a single center. The nonrandomized design might have affected our results, owing to unmeasured confounds, procedural bias, or detection bias. Despite the benefits of propensity matching, it is possible that there are additional confounds that were not accounted for in our adjustment algorithm. The whole study depends on the accuracy of propensity score matching and many pitfalls may be hidden. Second, it is generally believed that the experience of the surgeon can influence the results of OPCAB. Six experienced cardiac surgeons performed the OPCAB procedures in this study. However, our hospital is an international center for cardiovascular clinical and research. All of the surgeons followed the same standard OPCAB procedure of our hospital. Furthermore, this study was also limited to patients undergoing isolated OPCAB. Patients requiring concomitant cardiac surgical procedures, and/or those with mechanical complications of acute myocardial infarction, such as acute mitral regurgitation or myocardial rupture, were excluded from this study. Therefore, the results of this study cannot be extended to these extreme high-risk patient populations. Finally, the present study was conducted in the setting of a high-volume tertiary cardiovascular center in a developing country; therefore, the results might not be generalizable to other centers in different situations.

## Conclusions

In this large single center propensity score-matching analysis study, we showed that preoperative prophylactic IABP insertion in hemodynamic stability patients with severe LV dysfunction that underwent OPCAB was associated with reduced postoperative 30-day mortality, postoperative LCOS, and shorter postoperative LOS. Prospective, randomized, controlled trials are warranted to confirm these findings.

## Additional Information

**How to cite this article**: Yang, F. *et al*. Preoperative intra-aortic balloon pump improves the clinical outcomes of off-pump coronary artery bypass grafting in left ventricular dysfunction patients. *Sci. Rep.* **6**, 27645; doi: 10.1038/srep27645 (2016).

## Figures and Tables

**Table 1:** Clinical outcomes and complications of matched patients.

	**IABP** (**n** = **129**)	**Control** (**n** = **129**)	***p*****-value**
30-day mortality	2 (1.6%)	11 (8.5%)	0.02
Conversion to CABG	0 (0%)	6 (4.7%)	0.04
Number of distal anastomoses			0.22
Mean ± SD	3.1 ± 0.9	3.0 ± 0.9	
Median (IQR)	3 (3–4)	3 (2–3)	
Mechanical ventilation duration (h)			0.16
Mean ± SD	37.9 ± 40.9	47.1 ± 49.1	
Median (IQR)	24 (17–44)	26 (16–60)	
Prolonged mechanical ventilation (≥48 h)	27 (20.9%)	45 (34.9%)	0.02
IABP support time (h)			0.00
Mean ± SD	76.4 ± 33.4	135.9 ± 80.9	
Median (IQR)	56 (8–96)	128 (36–331)	
Length of ICU stay (h)			0.16
Mean ± SD	59.4 ± 50.4	72.6 ± 71.4	
Median (IQR)	48 (29–70)	48 (22–99)	
Postoperative LOS (d)			0.03
Mean ± SD	10.3 ± 5.2	12.7 ± 9.1	
Median (IQR)	9 (7–12)	10 (7–16)	
Hospital LOS (d)			0.17
Mean ± SD	21.4 ± 10.6	25.2 ± 15.2	
Median (IQR)	20 (13–27)	22 (14–32)	
Transfusion volume			
Packed red blood cells (units)			0.07
Mean ± SD	3.5 ± 4.8	4.9 ± 6.8	
Median (IQR)	2 (0–6)	2 (0–6)	
Fresh frozen plasma (ml)			0.37
Mean ± SD	638.0 ± 551.3	729.5 ± 681.5	
Median (IQR)	600 (400–800)	600 (400–800)	
Postoperative complications			
Postoperative low cardiac output	8 (6.2%)	18 (14%)	0.04
Postoperative myocardial infarction	11 (8.5%)	17 (13.2%)	0.23
Reoperation for bleeding	2 (1.6%)	7 (5.4%)	0.13
Tracheotomy	4 (3.1%)	8 (6.2%)	0.39
Renal failure requiring dialysis	0 (0%)	1 (0.8%)	1.00
Postoperative stroke	2 (1.6%)	1 (0.8%)	1.00
IABP-related complications			
IABP-related mortality	0 (0%)	0 (0%)	1.00
Limb ischemia requiring surgical intervention	1 (0.8%)	0 (0%)	1.00
Severe bleeding	0 (0%)	0 (0%)	1.00
Embolism	1 (0.8%)	0 (0%)	1.00
Balloon failure or leak	0 (0%)	0 (0%)	1.00

Continuous factors are summarized by median (25^th^ percentile, 75^th^ percentile) and categorical factors by frequency (percentage). IABP: intra-aortic balloon counterpulsation; ICU: intensive care unit; LOS: length of stay.

**Table 2:** Baseline characteristics before and after propensity-score matching.

	**Before matching**		***p-*****value**	**After matching**		***p-*****value**
	**IABP** (**n** = **191**)	**Control** (**n** = **225**)		**IABP** (**n** = **129**)	**Control** (**n** = **129**)	
Age (years)			0.05			0.27
Mean ± SD	57.7 ± 9.0	59.4 ± 9.5		59.1 ± 9.0	58.4 ± 9.3	
Median (IQR)	58 (52–64)	59 (53–66)		60 (54–65)	59 (51–66)	
Female	20 (10.5%)	34 (15.1%)	0.16	14 (10.9%)	12 (9.3%)	0.83
BMI (kg/m^2^)			0.06			0.84
Mean ± SD	25.1 ± 3.0	24.6 ± 3.3		25.0 ± 3.0	24.9 ± 3.3	
Median (IQR)	24.8 (23–27)	24.5 (22.5–26.4)		24.8 (22.8–27)	24.8 (22.7–26.9)	
LV diastolic dimension (mm)	63.3 ± 5.7	64.1 ± 5.9	0.12	63.4 ± 3.6	63.5 ± 3.4	0.83
LVEF (%)			0.26			0.94
Mean ± SD	32.1 ± 3.1	32.3 ± 3.2		31.9 ± 3.2	31.8 ± 3.5	
Median (IQR)	33 (30–35)	34 (31–35)		32 (30–35)	33 (30–34)	
NYHA class						
II	85 (44.5%)	81 (36%)	0.08	46 (35.7%)	48 (37.2%)	0.80
III	95 (49.7%)	131 (58.2%)	0.08	75 (58.1%)	74 (57.4%)	0.91
IV	11 (5.8%)	14 (6.2%)	0.84	8 (6.2%)	7 (5.4%)	1.00
Vessels diseased						
Left main coronary artery	20 (10.5%)	20 (8.9%)	0.59	13 (10.1%)	13 (10.1%)	1.00
Left anterior descending	185 (96.9%)	220 (97.8%)	0.56	126 (97.7%)	127 (98.5%)	1.00
Left circumflex artery	156 (81.7%)	167 (74.2%)	0.07	100 (77.5%)	98 (76%)	0.77
Right coronary artery	171 (89.5%)	196 (87.1%)	0.45	114 (88.4%)	113 (87.6%)	1.00
Triple-vessel disease	117 (61.3%)	121 (53.8%)	0.12	71 (55%)	68 (52.7%)	0.71
Unstable angina	140 (73.3%)	171 (76%)	0.53	97 (75.2%)	97 (75.2%)	1.00
Hypertension	86 (45%)	95 (42.2%)	0.57	55 (42.6%)	51 (39.5%)	0.61
Hypercholesterolemia	29 (15.2%)	20 (8.9%)	0.05	15 (11.6%)	16 (12.4%)	1.00
Diabetes mellitus	75 (39.3%)	71 (31.6%)	0.10	42 (32.6%)	38 (29.5%)	0.56
History of PCI	16 (8.4%)	32 (14.2%)	0.06	14 (10.9%)	16 (12.4%)	0.85
Chronic obstructive pulmonary disease	7 (3.7%)	10 (4.4%)	0.69	4 (3.1%)	4 (3.1%)	1.00
Peripheral vascular disease	11 (5.7%)	7 (3.1%)	0.19	4 (3.1%)	5 (3.9%)	1.00
Cerebrovascular disease	14 (7.3%)	22 (9.8%)	0.38	14 (10.9%)	10 (7.8%)	0.52
Recent (14 d) myocardial infarction	71 (37.2%)	55 (24.4%)	0.005	37 (28.7%)	41 (31.8%)	0.56
Mitral regurgitation	26 (13.6%)	23 (10.2%)	0.29	14 (10.9%)	16 (12.4%)	0.86
Previous myocardial infarction	133 (69.6%)	140 (62.2%)	0.11	87 (67.4%)	94 (72.9%)	0.33
Ventricular aneurysm	51 (26.7%)	100 (44.4%)	<0.001	42 (32.6%)	43 (33.3%)	0.90
Congestive heart failure	19 (10%)	15 (6.7%)	0.22	11 (8.5%)	10 (7.8%)	1.00
Preoperative renal insufficiency	38 (19.9%)	28 (12.4%)	0.04	23 (17.8%)	20 (15.5%)	0.73
Logistic EuroSCORE			0.91			0.96
Mean ± SD	6.8 ± 2.2	6.7 ± 2.1		6.8 ± 2.4	6.8 ± 2.3	
Median (IQR)	7 (5–8)	7 (5–8)		7 (5–8)	7 (5–8)	
Preoperative creatinine (μmol/L)			0.11			0.76
Mean ± SD	86.7 ± 22.6	83.3 ± 21.3		85.4 ± 18.7	84.2 ± 20.4	
Median (IQR)	80 (71.7–101)	79 (70.8–90)		80 (71.7–99)	79 (71.5–92)	
Preoperative lactate (mmol/L)			0.75			0.60
Mean ± SD	1.6 ± 0.7	1.6 ± 0.7		1.6 ± 0.7	1.6 ± 0.9	
Median (IQR)	1.5 (1.2–1.8)	1.5 (1.2–1.8)		1.5 (1.3–1.8)	1.5 (1.2–1.9)	
Preoperative CK-MB (mmol/L)			0.09			0.63
Mean ± SD	7 ± 25.5	3.1 ± 11.5		3.3 ± 11.1	4.3 ± 15	
Median (IQR)	1.5 (0.8–2.5)	1.3 (0.7–2)		1.4 (0.8–2.0)	1.4 (0.7–2.2)	
Preoperative cTNI (mmol/L)			0.10			0.53
Mean ± SD	3.1 ± 13.6	0.8 ± 3.9		0.7 ± 1.9	1.1 ± 5.0	
Median (IQR)	0.05 (0.03–0.70)	0.05 (0.02–0.20)		0.05 (0.03–0.18)	0.05 (0.03–0.50)	

BMI: body mass index; CK-MB: creatinine kinase isoenzyme MB; cTNI: troponin I; EuroSCORE: European System for Cardiac Operative Risk Evaluation; IABP: intra-aortic balloon pump; IQR: interquartile range; LV: left ventricular; PCI: percutaneous coronary intervention; SD: standard deviation.
